# Extracellular Vesicles from Adipose-Derived Mesenchymal Stem Cells Improve Ligament–Bone Integration After Anterior Cruciate Ligament Primary Repair in Rabbit

**DOI:** 10.3390/biom15030396

**Published:** 2025-03-10

**Authors:** Andre Yanuar, Hasrayati Agustina, Radiana Dhewayani Antarianto, Nucki Nursjamsi Hidajat, Andi Isra Mahyuddin, Ismail Hadisoebroto Dilogo, Nicolaas Cyrillus Budhiparama, Nur Atik

**Affiliations:** 1 Doctoral Program, Faculty of Medicine, Universitas Padjadjaran, Bandung 40161, Indonesia; andreyanuar@yahoo.com; 2 Department of Orthopaedic and Traumatology, Santo Borromeus Hospital, Bandung 40132, Indonesia; 3 Department of Pathology Anatomy, Faculty of Medicine, Universitas Padjadjaran/Hasan Sadikin General Hospital, Bandung 40161, Indonesia; hasrayati@unpad.ac.id; 4 Stem Cell and Tissue Engineering Research Cluster, Indonesian Medical Education and Research Institute, Faculty of Medicine, Universitas Indonesia, Jakarta 10430, Indonesia; radiana.dhewayani@ui.ac.id (R.D.A.); ismailortho@gmail.com (I.H.D.); 5 Department of Histology, Faculty of Medicine, Universitas Indonesia, Jakarta 10430, Indonesia; 6 Department of Orthopaedic and Traumatology, Faculty of Medicine, Universitas Padjadjaran/Hasan Sadikin General Hospital, Bandung 40161, Indonesia; nucki@unpad.ac.id; 7 Faculty of Mechanical and Aerospace Engineering, Institut Teknologi Bandung (ITB), Bandung 40132, Indonesia; aim@itb.ac.id; 8 Department of Orthopaedics and Traumatology, Faculty of Medicine, Universitas Indonesia/Cipto Mangunkusumo General Hospital, Jakarta 10430, Indonesia; 9 Stem Cell Integrated Medical Technology Service Unit, Faculty of Medicine, Universitas Indonesia/ipto Mangunkusumo General Hospital, Jakarta 10430, Indonesia; 10 Department of Orthopaedic and Traumatology, Faculty of Medicine, Universitas Airlangga, Surabaya 60132, Indonesia; 11 Department of Orthopaedics, Leiden University Medical Centre, 2333 ZA Leiden, The Netherlands; 12 Department of Biomedical Sciences, Faculty of Medicine, Universitas Padjadjaran, Bandung 40161, Indonesia

**Keywords:** ACL repair, adipose-derived MSC, extracellular vesicles, ligament–bone integration

## Abstract

Backgrounds: In this research, we want to find out whether extracellular vesicles (EVs) from adipose-derived mesenchymal stem cells (MSCs) can improve ligament–bone integration after primary Anterior Cruciate Ligament (ACL) repair by performing immunological and biomechanical tests. Methods: All of the rabbits underwent ACL resection at the proximal attachment to the femur bone, and then were divided into four groups. We performed an ELISA examination from the tissue at the bone–ligament interface of iNOS, CD206, MMP-3, and TIMP-1 to evaluate their levels at the inflammatory stage at the end of the first week. Immunoexpression of type I and III collagen and failure load biomechanical tests were performed at the end of the sixth week. Result: The group that underwent ACL repair with EVs augmentation had significantly higher levels of CD206, significantly lower MMP-3 levels, and significantly higher TIMP-1 levels in the first week. The iNOS levels in the group that underwent ACL repair with EVs augmentation were significantly different compared to the control group that did not receive any. The number of type I collagen fibers and the failure load levels in the group that underwent ACL repair with EVs augmentation were significantly higher. Conclusions: EVs from adipose-derived MSCs can improve the outcome of primary ACL repair in rabbits by regulating the inflammatory process during the healing period.

## 1. Introduction

The Anterior Cruciate Ligament (ACL) is the most frequently injured ligament in the knee and accounts for more than 50% of knee injuries [1]. ACL reconstruction has become the gold standard in the treatment of unstable ACL injuries; however, the result of this procedure is imperfect. Neurological reconstitution cannot be achieved completely with ACL reconstruction [2,3,4], and about 45% of patients who undergo ACL reconstruction surgery experience early osteoarthritis within 10 years [5]. Before ACL reconstruction surgery became standard, orthopedic surgeons performed a repair procedure, but, because the results were not satisfactory, ACL reconstruction was still an option rather than ACL repair [6,7,8]. A meta-analysis involving 2401 patients from 28 studies showed that ACL reconstruction still had better survivorship compared to ACL repair [9].

ACL repair has several theoretical advantages compared to ACL reconstruction, such as preserved proprioceptive and kinematic function, which means maintaining neurological function in the knee, the preservation of bone stock, and reduced morbidity related to tissue donors for ligament grafts, because ACL repair does not require a tendon graft [10,11].

The success of an ACL operation is determined by the healing process between the ligament and the tunnel in the bone [8,12]. Following ACL reconstruction surgery and tendon graft implantation, the first cellular response is the gradual build-up of inflammatory cells, particularly neutrophils and M1 macrophages, which eventually transform into M2 macrophage cells [1,13,14].

Another challenge of the ACL–bone integration process is its intra-articular location, where the ligament tissue is submerged in synovial fluid, making the healing process more difficult compared to extra-articular ligaments [8]. Matrix metalloproteinases (MMPs), lysyl oxidases (LOXs), and tissue inhibitor of metalloproteinase 1 (TIMP-1) were detected in the synovial fluid of patients with ACL rupture. These MMPs can affect the microenvironment in the joints; therefore, the cartilage, meniscus, and posterior cruciate ligament (PCL) can also be affected and become damaged, and cause osteoarthritis [15,16].

In the proliferation phase, abundant but irregular collagen matrix deposition occurs, mainly composed of type III collagen, which resembles Sharpey-like fibers [17]. In the remodeling phase, type III collagen will gradually be replaced by type I collagen. Sharpey-like fibers increase gradually from 6 to 12 weeks. These Sharpey-like fibers are an important biomechanical factor that determines the biomechanical strength of the tendon graft/ligament–bone integration after ACL surgery [17,18].

Many strategies have been implemented to speed up and enhance the quality of tendon/ligament–bone integration using drugs, growth factors, platelet-rich plasma (PRP), stem cells, conditioned media, biomaterials, and biophysical interventions. Among these strategies, the use of mesenchymal stem cells (MSCs) has attracted a lot of research interest and has been extensively used in tissue engineering because of its capacity to proliferate, repair itself, and differentiate into various types of tissue [8,12,14,15,19]. However, although the use of stem cell therapy for tissue engineering and regeneration is very promising, due to storage limitations, and because cell aging occurs during in vitro expansion, the application of stem cells in clinical practice is difficult to implement. Recent research shows the mechanism by which stem cells repair tissue through a paracrine pathway, which can be carried out through extracellular vesicle (EVs)-derived MSCs [20,21,22,23].

Extracellular vesicles (EVs) are nanovesicles that have a lipid bilayer membrane structure that is owned by MSCs. EVs have a diameter of 30–200 nm and contain a variety of molecules, including proteins, lipids, DNA, mRNA, lncRNA, miRNA, and circular RNA. Among them, EVs miRNAs are the most numerous EVs cargo molecules. The miRNAs produced by EVs can mediate the paracrine and endocrine pathways between tissues to regulate gene expression [24]. They are involved in the exchange of information and substances between cells, and can stimulate the regeneration and repair of tissues and organs, including the heart, skin, liver, cartilage, tendons, and ligaments. The use of cell-free therapies, such as the use of EVs derived from MSCs, can provide more benefits than the use of MSCs themselves, such as the absence of immune reactions to stem cells and the reduced risk of thromboembolism, and can be evaluated similarly to pharmacological agents [20,21,22,25,26]. The effectiveness of MSC-derived EVs in improving the quality of tendon/ligament integration into bone occurs due to the protein content, which can regulate the polarization of M1 macrophages and M2 macrophages [21,27,28]. EVs produced by adipose-derived MSCs have also been shown to increase the expression of the Col-1a1 gene, which stimulates fibroblasts to produce type I collagen [29]. EVs have also been shown to be able to modulate the matrix by reducing MMP-3 expression and increasing TIMP expression [19,30,31].

To improve the ligament–bone integration following ACL primary repair, we augmented EVs from adipose-derived MSCs in the tunnel at the site of the bone–ligament interface. The purpose of this study was to evaluate whether the augmentation of EVs from adipose-derived MSCs could improve the ligament–bone integration following ACL primary repair. M1 macrophages, M2 macrophages, MMP-3, TIMP-1, collagen type I and III expression, and biomechanical tests were used to examine the outcomes. Since miRNAs constitute an important fraction of the EVs content and are key contributors to the biological function of EVs [32], we also performed miRNA profiling in the EVs that were upregulated and downregulated after being augmented in rabbits.

## 2. Materials and Methods

### 2.1. Study Design

**Chart 1:**
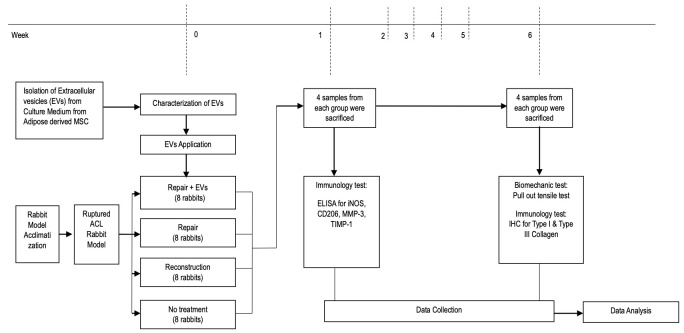
Outline of the research timeline.

New Zealand white rabbits (Oryctolagus cuniculus) were used for the randomized animal laboratory research. The Padjadjaran University Animal Experiment Ethics Committee authorized the study procedure (No. 380/UN6.KEP/EC/2023). The animals received resection in the proximal attachment to the femoral bone (type I ACL rupture) [33], and then were randomly divided into the following four groups: (1) group I, control I group (*n* = 8), which received no intervention; (2) group II, control II group (*n* = 8), which received an ACL reconstruction surgery; (3) group III, treated I group (*n* = 8), which received an ACL repair surgery; (4) group IV, treated II group (*n* = 8), which received an ACL repair surgery with EVs augmentation. Half of the number of each group was evaluated using the tissue at the tendon/ligament–bone interface at the end of first week for the enzyme-linked immunosorbent assay (ELISA) examination of iNOS (representing the M1 macrophage), CD206 (representing the M2 macrophage), MMP-3, and TIMP-1 in order to evaluate their levels at the inflammatory stage of the healing process (subgroup A). The rest of the animals were sacrificed at the end of the sixth week for the immunoexpression (type I and III collagen) and failure load biomechanical tests. The immunoexpression test used the right hind leg, while the biomechanical test used the left hind leg (subgroup B). The outline of the entire research process is presented in Chart 1.

**Figure 1:**
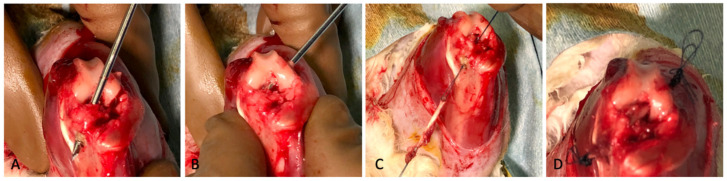
(**A**) Tunnel preparation; 1.8 mm Kirschner wires were drilled at the anatomical insertions of the ACL in the tibia (**B**) and in the femur (**C**). The tendon graft was passed through the tunnels with the help of needle 23 (**D**). The tendon graft ends under tension with their sutures; knots were made up to 6 times in the tibial dan femoral side until the size of the knot exceeded the diameter of the bone tunnel and resembled an endobutton implant.

**Figure 2:**
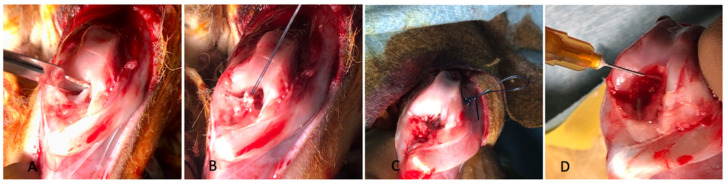
(**A**) Transection of the ACL attachment at the medial site of the lateral femoral condyle. (**B**) Suturing at the edge of the ligament graft using the Krackow technique. (**C**) The suture was passed through the femoral tunnels with the help of needle 23, and knots were made until the size of the knot exceeded the diameter of the bone tunnel. (**D**) Injection of EVs at bone-ligament interface.

The surgeries were carried out by four individuals, including one technician, two assistant surgeons, and one researcher. Eight rabbits underwent bilateral ACL reconstruction using an extensor digitorum longus tendon graft. The ACL reconstruction procedure was performed on the rabbits under general anesthesia, administered gradually by intramuscular injections of 20 mL of Ketamine for each rabbit. Following the removal of the ACL, tibial, and femoral bones, tunnels were created (Figure 1A,B) to implant the graft at the ACL native position. For the best ACL reconstruction isometry (isometric angle), Kirschner wires (1.8 mm in diameter) were drilled at the anatomical insertions of the ACL (isometric angle). To achieve loading conditions like those of the native ACL, the drill holes had to be set consistently and properly in reference to the original ACL insertion points. The tendon graft was passed via the tibial tunnels before continuing through the femoral tunnel with the help of needle 23. For the tendon graft ends under tension with their sutures, knots were made up to six times, until the size of the knot exceeded the diameter of the bone tunnel and resembled an endobutton implant (Figure 1C,D). The ACL reconstruction surgical technique in the rabbits was in accordance with the recommendations of Bachy et al. [34]. Sixteen rabbits underwent bilateral ACL repair after the original ACL was resected in the proximal origin in the lateral femoral condyle. The procedure was the same as with the ACL reconstruction, except that a tendon graft was not used in the ACL repair; rather, the ends of the ligament were sutured directly using Monofilament Polyamide 3.0 sutures with the Krackow technique, and the thread was passed through the tunnel to be fixed to the lateral femur. Eight of those rabbits received 100 μL of EVs injected into the femoral tunnel at the bone–ligament interface (group IV) (Figure 2). The dose given, 40 μg/100 μL, referred to previous research [35]. Based on the literature, the average length of the femur was 9 cm (90 mm) [36]; therefore, it was estimated that the length of the femoral tunnel to be made was 20 mm, while the diameter to be made was 2 mm, so the tunnel volume should be 62.8 mm^3^ or 62.8 μL. By calculating the administration to the ligament stump and tunnel, a dose of 100 μL was considered to be a sufficient application. After the procedure, the animals were allowed to move freely around in their cages.

### 2.2. Isolation, Culture, and Characterization of Adipose-Derived MSCs (Ad-MSCs)

Adipose-derived MSCs were obtained from cryo-Ad-MSCs stored in liquid nitrogen tanks using the method described in previous research [37]. The medium for the MSCs was composed of 10% platelet-rich plasma (PRP), 1% amphotericin B (250 µg/mL), 1% pen-strep (10,000 U/mL of penicillin), 1% heparin (1000 U/mL), 1.000 µg/mL of streptomycin, and α Minimum Essential Medium (MEM). A total of 9 mL of complete medium was added to the centrifuge tube with 1 mL of cells from the cryotube. Afterwards, the pellets were separated from the supernatant, and then centrifuged at a speed of 177× *g* for 10 min. Then, the cells were cultured at a density of 5 × 10^3^ cells per cm^2^. Every experiment was carried out using 6 culture passage cells. Using flow cytometry, the Ad-MSCs were identified in accordance with the International Society of Cellular Therapy’s (ISCT) standards [38].

### 2.3. Isolation and Characterization of Extracellular Vesicles

Extracellular vesicles were isolated from the culture medium (CM) from the Ad-MSCs that reached 70% confluency according to method previously described [37]. The frozen CM was defrosted by immersing it in room-temperature water. The CM solution was centrifuged at a speed of 750× *g* at 20 °C for 15 min. The supernatant was collected, and then it was further centrifuged at a speed of 2000× *g* for 15 min using the Eppendorf Centrifuge 5804R. Then, the supernatant was collected and spinned at 10,000× *g* for 45 min using the Thermo Scientific Heraeus Fresco 17 Microcentrifuge. The supernatant was collected and filtered using a 0.2 μm syringe filter; then, ultracentrifugation with a speed of 100,000× *g* at 4 °C was performed for 90 min using the Thermo Scientific Sorvall WX+Ultra Series Ultracentrifuge.

The EVs-containing pellets were then transferred to a 15 mL falcon tube after the supernatant was discarded. After adding cold D-PBS until the volume reached 5 mL, the mixture was re-dissolved. After that, the EVs were separated into 1 mL cryovials and stored for one year in either a freezer at −80 °C or a cryo-box chiller at −20 °C. Using a Horiba SZ 100z machine, which can also detect the suspension sample’s zeta potential, the size and distribution of the EVs were assessed using a particle size analyzer (PSA). The zeta potential was performed in triplicate for each experiment at 25 °C.

### 2.4. ELISA Evaluation for iNOS, CD206, MMP-3, and TIMP-1

The levels of iNOS, CD206, MMP-3, and TIMP-1 from the tissue at the bone–ligament/tendon interface at one week following surgery were examined using the ELISA method. This examination was carried out with a double-antibody sandwich technique using monoclonal antibodies as the capture antibodies and biotin-conjugated polyclonal antibodies as the detection antibodies for the MMP-3, TIMP-1, iNOS (Cloud-Clone Corp, Houston, TX, USA), and CD 206 (Wuhan Feiyue Biotechnology Co., Ltd, Wuhan, China).

### 2.5. Immunoexpression Assessment of the Tendon/Ligament–Bone Interface

To evaluate osteointegration at the tendon/ligament–bone interface 6 weeks following surgery, we performed immunostaining for the collagen type I and III quantification. Immunohistochemistry was performed using the labeled streptavidin biotin immunoperoxidase complex method with the Starr trek universal HRP detection system (Biocare Medical, Pacheco, CA, USA). The monoclonal primary antibodies included were collagen I (Gene Tex, Irvine, CA, USA) and collagen III (Gene Tex, USA). The procedure used for the immunohistochemistry was as follows. A conventional histology hotplate was used to heat 4µ thick slices for one hour at 60 °C after they were cut onto glass slides coated with 0.01% poly-L-lysine. The sections were treated with three variations in ethanol and alcohol after being dewaxed in xylene, and then were submerged in water. The sections were heated to induce antigen retrieval in citrate buffer (pH of 6.0) for 15 min in a decloaking chamber (DC2008INTL, Biocare Medical, USA). This was followed by 20 min of cooling at room temperature. The sections were then incubated for an hour at room temperature, treated to prevent endogenous peroxidase, and stained with primary antibodies. The 3,30 diaminobenzidine chromogen, a horseradish peroxidase polymer-based detection system (Biocare Medical), and hematoxylin counterstain were used for the detection process. The same conditions were applied to all staining techniques, and the sections that were representative of the tendon/ligament–bone interface were chosen for the assessment of the osteointegration.

Image acquisition was performed using an Olympus CX41 trinocular microscope, an Olympus X0.5 camera mount, and an H300 microscope camera. The magnification of the objective camera was 40×. The acquired microscopic image size was 1.920 × 1.080, with 24-bit RGB 3 channels. The images were compressed by the lossless Tagged Image File Format (TIFF) algorithm. The acquired images were preprocessed to maintain the homogeneity of the color distribution and intensity. The staining algorithm used the deconvolution from Ruifrok et al. [39]. The segmentation of the image into regions was then carried out. This image analysis process included thresholding, edge detection, and watershed segmentation. Measurements were taken from all of the image segments. The measurements consisted of the segment longitudinal length and segment transverse length. Quantitative analysis of the collagen was carried out with the help of ImageJ software ver. 1.54d by looking at the mean longitudinal and transversal lengths of the fibers.

### 2.6. NanoString MicroRNA Profiling

A wide range of miRNAs in ligament injury have been shown to play vital roles in maintaining and regulating its physiological function. In this study, as additional information on the benefits of EVs, we performed miRNA profiling of the EVs samples used for this research, and analyzed how they were regulated in the first and sixth weeks.

The expression of the miRNAs in the EVs and the samples from the animals was evaluated using NanoString Technology (Genetika Science, Tangerang, Indonesia). In situ hybridization (ISH) probes or oligonucleotide barcodes linked with antibodies were used by the NanoString to measure the RNA or protein expression in two or three dimensions. Total RNA extraction was carried out according to the Zymo kit instructions. RNA samples were run on the nCounter^®^ miRNA Expression Panel. The increase or decrease in the miRNA expression was compared between the groups of rabbit ligaments at six weeks following repair (Cluster 3) and human EVs (Cluster 1), and between the groups of rabbit ligaments at one week following repair (Cluster 2) and human EVs (Cluster 1).

### 2.7. Biomechanical Testing

Finally, to assess the bone–ligament/tendon integration, we evaluated the failure mode. All specimens in all groups failed by the pull-out tensile test at six weeks postoperatively. Six weeks is the beginning of the proliferation phase of the healing process.

Immediately following sacrifice, the specimens were prepared for the biomechanical testing. Confounding influences were eliminated by meticulously removing all of the soft tissue before testing, except for the graft in the knee cavity and the suture at the tunnel exits. An apparatus for material testing (Instron 5985, USA) was clamped to hold the femur–graft–tibia complex in place using specially made iron tubes. The tensile load rose steadily at a rate of 10 mm per minute of displacement. Using Bluehill Universal software, the displacement and tensile load were recorded on the load–deformation curve and analyzed.

### 2.8. Statistical Analyses

Data obtained from the ELISA examination (iNOS, CD206, MMP-3, and TIMP-1 levels), immunohistochemistry examination (longitudinal and transverse lengths of type I and III collagen fibers), and biomechanical tests (failure load test and ΔL at failure load) were tested for the distribution and homogeneity using the Shapiro–Wilk and Levene tests. All of quantitative data are expressed as the mean ± standard deviation (SD) for the normal distribution data and expressed as the median with interval range for the non-normal distribution data. For the data with a normal distribution and homogeneous data, a one-way ANOVA comparison test was carried out, followed by post hoc LSD, while, for the data with a non-normal and inhomogeneous distribution, an alternative Kruskal–Wallis test was carried out, followed by a post hoc Mann–Whitney test. Here, *p* < 0.05 was considered to indicate a significant difference. Data analysis was performed using SPSS version 26.

## 3. Results

### 3.1. Characterization of Ad-MSCs

**Figure 3:**
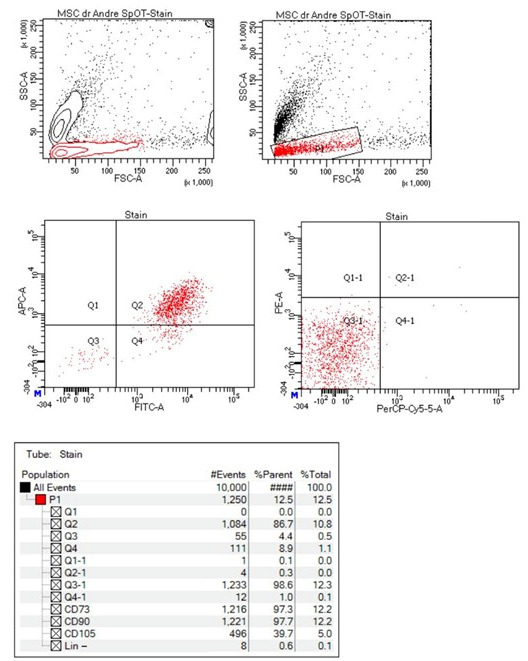
Characterization using flow cytometry showing that 97.3% of the population expressed CD73 and 97.7% expressed CD90.

The isolated Ad-MSCs were a homogeneous cell population, and the phenotypes were characterized by immunocytochemistry: about 97.3% of the Ad-MSCs showed the positive expression of CD73, and 97.7% showed the positive expression of CD90 (Figure 3).

### 3.2. Characterization of the Extracellular Vesicles of Ad-MSCs

The EVs were successfully isolated from the Ad-MSCs using the ultracentrifugation technique. The mean size of the EVs was 146.6 nm ± 76.3 nm. The distribution pattern was normal and had a mode value of 111.4 nm. The stability of the EVs was determined by measuring the zeta potential. The zeta potential of all EVs preparations was negative and distributed within the range of −2.1 mV to −4.6 mV. The conductivity was 12,698 mS/cm. Several tetraspanins, including CD63, CD81, and CD82, were used for the markers of the EVs, and at least one protein in category 1 had to be analyzed so to demonstrate the EVs nature and the degree of purity of an EVs preparation [40].

**Figure 4:**
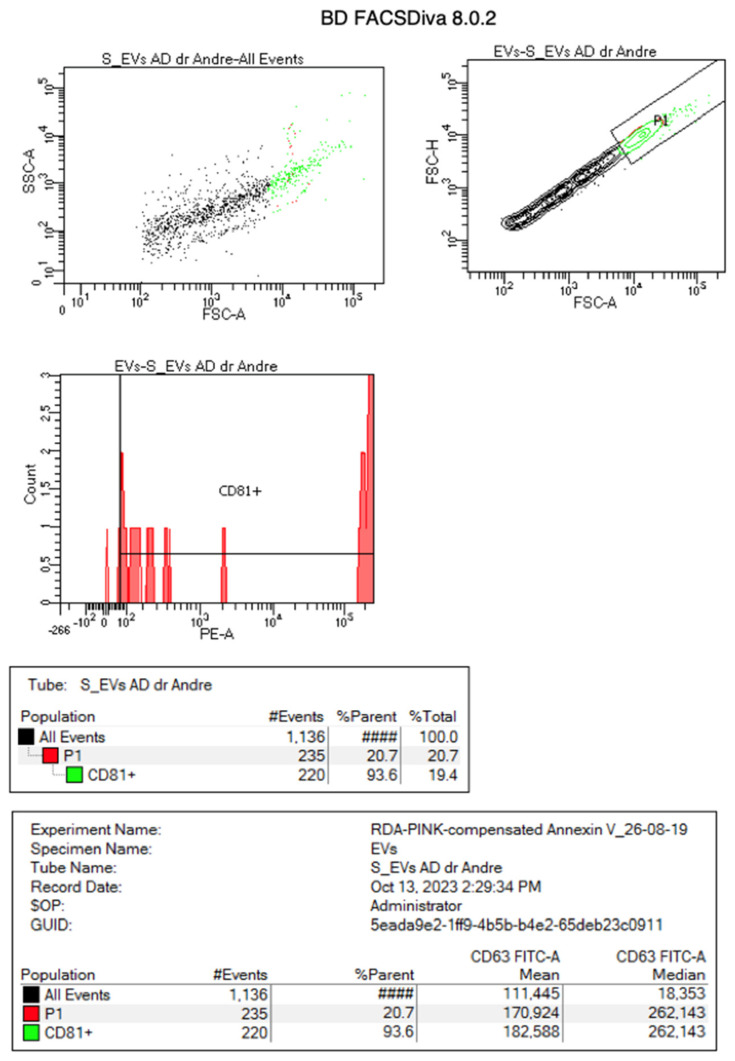
Flowcytometry test showing that 93.6% of the adipose-derived MSC EVs had a positive CD81+ expression.

To check the purity of the EVs, CD81+ expressions were evaluated using the protocol from BD Sciences. About 93.6% of the EVs showed the positive expression of CD81+ (Figure 4).

### 3.3. Evaluation of the Levels of iNOS, CD206, MMP-3, and TIMP-1

The levels of the iNOS, CD206, MMP-3, and TIMP-1 obtained from the results of the ELISA examination of the ligament/tendon tissue samples at the ligament/tendon–bone interface at the end of first week are presented in Table 1. Prior to carrying out the comparative analysis, all of the data were tested to determine their distribution and homogeneity. The data on the CD206 levels were not normally distributed and had a homogeneous variance. The data on the iNOS levels were normally distributed and the homogeneity of the variance was heterogeneous. The data on the MMP-3 levels were not normally distributed and had a homogeneous variance. The TIMP-1-level data had a normal distribution and a homogeneous homogeneity. From Table 1, it is shown that the MMP-3 and TIMP-1 levels had significant differences between the groups (*p* = 0.012 and *p* = 0.029). Next, a post hoc statistical test was carried out to determine whether there were differences between each group in each variable marker. The Mann–Whitney post hoc test showed that the levels of CD206, which represents the M2 macrophages in the treated II group, had a significantly higher median value compared to the treated I group (*p* = 0.029). The levels of iNOS, which represents the M1 macrophages in the treated II group (group IV), had a smaller median value compared to the treated I group (group III), but this difference was not statistically significant; however, the Mann–Whitney post hoc test showed that it had a statistically significant difference from the control I group (*p* = 0.029). The levels of MMP-3 in the treated II group had a significantly lower median value compared to the treated I group (*p* = 0.029). The levels of TIMP-1 in the treated II group had a significantly higher mean value compared to the treated I group (*p* = 0.030).

### 3.4. Immunoexpression Analysis of Type I Collagen Fibers and Type III Collagen Fibers

**Figure 5:**
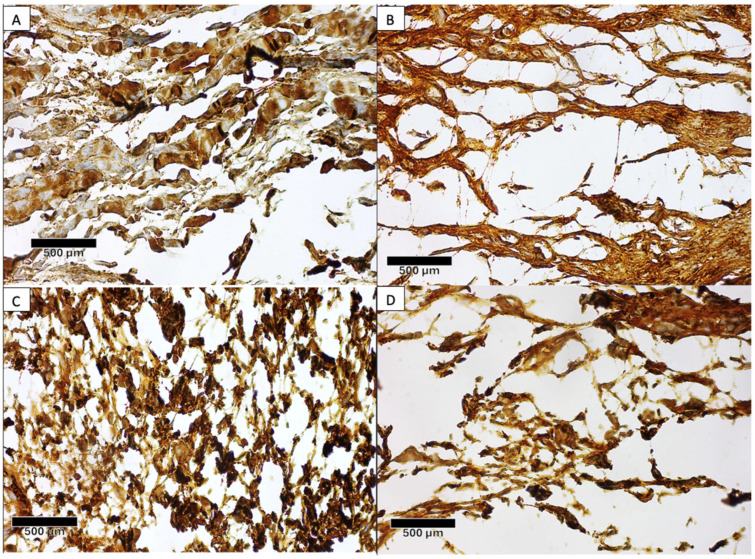
The groups that received EVs augmentation had longer longitudinal (**A**) and transversal (**C**) collagen type I fiber lengths compared to the longitudinal (**B**) and transversal (**D**) fiber lengths in the control group.

The immunoexpression analysis of type I collagen fibers from the tissue samples obtained from the tendon/ligament–bone interface at week six is shown in Table 2. Prior to carrying out the comparative analysis, all of the data were tested to determine their distribution and homogeneity. Data on the longitudinal length of type I collagen were normally distributed, and the homogeneity was heterogeneous. Data on the transverse length of type I collagen were not normally distributed, and the homogeneity was homogeneous. From Table 2, it is shown that both the longitudinal and transverse length of type I collagen from all groups had significant differences. The treated II group had the highest median value of longitudinal and transverse fiber lengths for type I collagen fibers compared to the other groups (Figure 5); however, the Mann–Whitney post hoc test showed that it had the most significant difference with the control I group (*p* = 0.029).

The immunoexpression analysis of type III collagen fibers are shown in Table 3. Data on the longitudinal length of type III collagen were normally distributed and homogeneous. Data on the transverse length of collagen type III were normally distributed and homogeneous. The research result showed that, between the study groups, the mean values were almost the same and did not show any significant differences between the groups (*p* > 0.05).

### 3.5. miRNA Profiling

The RNA samples were run on nCounter^®^ miRNA Expression Panels. The panel utilizes NanoString technology to perform expression profiling by direct the quantification of individual RNA molecules. The nCounter miRNA Expression Panels analyzed up to 827 human miRNAs. The data obtained from the run were analyzed using nSolver Analysis Software 4.0 and the ROSALIND platform. To make the analysis of miRNA profiling easier, we created three analysis clusters.

The differential expression of the miRNAs was observed in Cluster 1 (EVs) versus Cluster 2 (rabbit ligaments at one week following repair) versus Cluster 3 (rabbit ligaments at six weeks following repair). Out of the 827 miRNAs assessed, 24 had significantly increased and 18 had significantly decreased expressions in the one-week-following-repair group. The top 10 upregulated miRNAs in Cluster 2 compared to Cluster 1, sorted according to the *p*-values from the smallest to the largest, are as follows: miR-6503-3p, miR-22-3p, miR-517c-3p + miR-519a-3p, miR-134-5p + miR-6728-5p, miR-1302, miR-608, miR-105-5p, miR-152-3p, and miR-16-5p. The top 10 downregulated miRNAs in Cluster 2 compared to Cluster 1, sorted according to the *p*-values from the smallest to the largest, are as follows: miR-374a-5p, miR-548h-5p, miR-147a, miR-106b-5p, miR-135b-5p, miR-450b-3p, miR-135b-5p, miR342-5p, miR148a-3p, miR-876-5p, and miR-507.

Further assessment showed that 11 of the miRNAs had significantly increased and 6 of the miRNAs had significantly decreased expressions in the six-weeks-following-repair group. The upregulated miRNAs in Cluster 3 compared to Cluster 1, sorted according to the *p*-values from the smallest to the largest, are as follows: miR-4531, miR-3180-5p, miR-510-3p, miR-3065-3p, miR-628-3p, miR-30e-5p, miR-219a-2-3p, miR-30a-5p, miR-548 y, and miR-324-3p. The downregulated miRNAs in Cluster 3 compared to Cluster 1, sorted according to the *p*-values from the smallest to the largest, are as follows: miR-512-5p, miR-26a-5p, miR-641, miR-874-3p, miR-1288-3p, and miR-1268b.

### 3.6. Biomechanical Analysis

**Figure 6:**
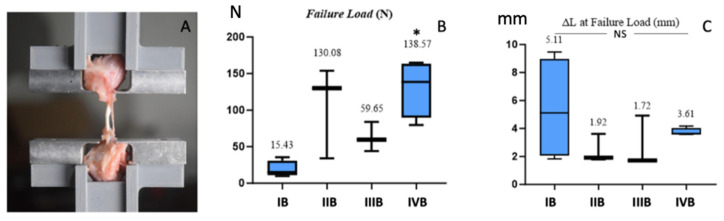
The femur–ligament–tibia complex was fixed in a custom iron device and clamped to an Instron machine for the biomechanical testing (**A**). The treated group with EVs augmentation had the greatest failure load force, * indicates a statistically significant difference (**B**), whereas the control group without any treatment had the most elongation before failure; however, this difference was not statistically significant (NS: Not Significant) (**C**).

In the evaluation of the failure mode, all specimens in all groups failed by the pull-out tensile test at six weeks postoperatively. The failure load test data showed a normal distribution and a heterogeneous homogeneity. The data on the increase in the ligament/tendon lengths showed that they were not normally distributed and had a heterogeneous homogeneity. The increase in the ligament/tendon length just before reaching the maximum force was also measured and is expressed in ΔL at the failure load (Figure 6). The treated II group had the greatest failure load, followed by the control II group, the treated I group, and the control I group. These four groups showed significant differences between each other (*p* = 0.033), but the Mann–Whitney post hoc test showed that the most significant difference was found in the treated II group compared to the control I group (*p* = 0.029). The greatest increase in length was found in the control I group, followed by the treated II group, the control II group, and the treated I group. However, this difference was not statistically significant (*p* > 0.05).

### 3.7. Correlation Between CD206, MMP-3, TIMP-1, and Collagen Type I and Failure Load

**Figure 7:**
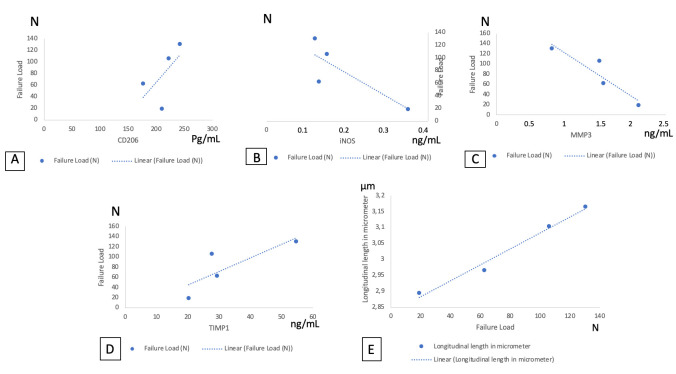
(**A**) Correlation between CD206 and the failure load. (**B**) Correlation between iNOS and the failure load. (**C**) Correlation between MMP-3 and the failure load. (**D**) Correlation between TIMP-1 and the failure load. (**E**) Correlation between the longitudinal length of type I collagen and the failure load.

In this study, we also wanted to know the relationship between the variables that play a role in the healing process on the strength of the failure load test. We found a very strong correlation between MMP-3 and the failure load (*p* = 0.074; r = −0.926), TIMP-1 and the failure load (*p* = 0.197; r = 0.813), CD206 and the transverse length of type I collagen fibers (*p* = 0.121; r = 0.879), MMP-3 and the transverse length of type I collagen fibers (*p* = 0.173; r = −0.827), MMP-3 and the longitudinal length of type I collagen fibers (*p* = 0.096; r = −0.904), and the failure load and longitudinal length of type I collagen fibers (*p* = 0.009; r = 0.991). However, the only statistically significant correlation was between the longitudinal length of type I collagen fibers and the failure load (Figure 7).

## 4. Discussion

The connection between ligaments and bones is a complex and diverse junction involving bone, mineralized fibrocartilage, non-mineralized fibrocartilage, and ligaments. This structure plays an important role in passing on energy from one bone to another and in preventing the accumulation of excessive energy. Changing the normal composition of this transition area will certainly reduce the biomechanical strength [41]. The healing process that integrates ligaments to bone (ligament–bone healing), as in ACL repair procedures, and tendons to bone, as in ACL reconstruction procedures, is still a challenge in the field of orthopedics and sports medicine [17]. This healing process begins with an inflammatory phase involving the important role of macrophage cells. The first cellular response immediately after ACL reconstruction surgery with tendon graft implantation is the continuous accumulation of inflammatory cells, mainly neutrophils and M1 macrophages, with a progressive transition into M2 macrophage cells [1]. Animal models that enhance the M2 macrophage activity in the healing tendon have shown reduced scar formation, accelerated healing, decreased inflammation, and even increased biomechanical strength [13], while EVs are known to polarize M1 macrophages into M2 macrophages. EVs derived from adipose mesenchymal stem cells (Ad-MSCs), among the numerous forms of regenerative medicine, provide the best chances for tendon regeneration [21]. In our study, we found that the group given EVs derived from Ad-MSCs had higher levels of M2 macrophages than the control group. This finding is in line with the expression of miRNA-26-5p, which was downregulated six weeks after ACL repair compared with its expression in the EVs that we isolated. Yu et al., in previous study, found that miR-26a inhibited prostaglandin E2 (PGE2) production by targeting cyclooxygenase-2 (COX-2), thus decreasing the inflammatory response. They found that miR-26a was downregulated in vitro and in vivo. The miR-26a mimic significantly reduced the COX-2 protein levels, further inhibiting the proinflammatory cytokine production in LPS-stimulated macrophages. Their findings demonstrated that miR-26a inhibited COX-2 expression, implicating miR-26a as a drug target for the progression of inflammation [42]. The downregulation of miR-26a also facilitated the upregulation of KLF4, which, in turn, favored increased arginase and decreased iNOS activity, thus favoring M2 polarization [43].

In addition, the content of miR-374a-5p in Ad-MSC-derived EVs may be related to the regulatory process of inflammatory cells, as found by Sanchez et al., who explored the role of miR-374a-5p in the pathogenesis of inflammatory bowel disease (IBD) and identified the reduced expression of miR-374a-5p in IBD monocytes, which correlated with a module of upregulated genes related to the inflammatory response. The main proinflammatory module genes, including, for example, *TNFα*, *IL1A*, *IL6*, and *OSM*, were inversely correlated with miR-374a-5p [44]. Li et al. discovered that downregulating miR-374a-5p could inhibit M1 macrophage polarization [45].

Matrix metalloproteinase (MMP) enzyme activity, produced by fibroblasts, was detected in the synovial fluid following an ACL rupture. These MMPs can affect the microenvironment in the joints, so that the cartilage, meniscus, and posterior cruciate ligament (PCL) can also be affected and become damaged, leading to osteoarthritis [15,16]. The higher the MMP/TIMP ratio, the greater the risk of cartilage tissue damage [15]. This enzyme imbalance affects the ACL healing process [15,46]. Higuchi et al. identified that, after ACL rupture, the concentration of MMP-3 remained high, regardless of the time interval after the ACL rupture [47]. Wang et al., in a previous study, found that EVs from tendon stem cell injections significantly reduced MMP-3 levels, increased the expression TIMP-3 and Col-1a1 levels, and improved the biomechanical abilities of the ultimate stress and maximum loading [31].

Our study proves that EVs derived from Ad-MSCs can also reduce MMP-3 levels and increase TIMP-1 levels. This is likely related to the expression of miR-874-3p in the EVs we isolated. Yang et al., in previous research, also found that the expression of miR-874-3p was associated with a decrease in MMP-3 levels in the nucleus pulposus in the intervertebral disk [48]. Another advantage of controlling MMP-3 levels is the reduced risk of osteoarthritis after ACL surgery, as is currently the case, where 45% of postoperative ACL reconstruction patients experience OA after 10 years [5].

Matrix metalloproteinases (MMPs) and their natural inhibitors, tissue inhibitors of metalloproteinases (TIMPs), are central to the regulation of the ECM. Studies from Huang et al. have shown that miR-145 can increase TIMP-1 expression by targeting a negative regulator of TIMP-1, thereby promoting its production [49]. However, in this research, we were not able to isolate the presence of miR-145 from the EVs that we isolated, so it seems that there is a role for other miRNAs that influence macrophage polarization, and that MMP-3 plays an indirect role in increasing TIMP-1 levels.

Entering the proliferation phase, abundant but irregular collagen matrix deposition occurs, mainly composed of type III collagen, which resembles Sharpey-like fibers. The newly synthesized collagen matrix restores bone–ligament continuity. In the remodeling phase, type III collagen will gradually be replaced by type I collagen starting at week six. These Sharpey-like fibers are an important biomechanical factor that determines the biomechanical strength of the tendon graft in the bone tunnel after ACL surgery [17,18].

We examined the number of collagen fibers at the ligament/tendon–bone interface between all groups at week six, and the group that received EVs augmentation had a significant number of type I collagen fibers compared to the other groups. The number of type I collagen fibers is described by the longitudinal length and transverse length, both of which provided a statistically significant difference from the control group. This large number of type I collagen fibers seemed to be related to the low levels of MMP-3 [50].

In this study, we also found a significant correlation between the longitudinal length of type I collagen fibers and the failure load, which represents the quality of integration between ligaments/tendons and bone. This finding is in line with the fact that well-controlled inflammation from the beginning of the healing process will result in a good quality integration between the ligaments/tendons and bone. A prolonged inflammatory reaction will disrupt the repair process because it will produce many scars. The ligament scar is mainly composed of type III collagen, which is structurally, chemically, and mechanically abnormal even on long-term follow-up. The amount and type of collagen in the scar area is different from the intact ligament because the normal collagen content decreases by 70% and the amount of type III collagen increases significantly [17]. In our study, the highest number of type III collagen fibers was found in the group that underwent ACL repair without EVs augmentation, but it was not significantly different from the other groups.

The key to successful ACL surgery is a good quality connection between the tendon/ligament and the bone. The ligament contact area with bone in ACL repair is less than the tendon contact area with bone in ACL reconstruction. A really good quality connection resembles the origin expected to occur in this small area of connection between the bone and ligament by controlling the inflammatory process from the very beginning of the healing process. The EVs derived from the Ad-MSCs seemed to be successful in regulating the inflammatory process, since macrophage polarization controlled the MMP-3 levels, which ultimately had an impact on the production of more type I collagen fibers, such that the failure load in the group repaired with EVs augmentation was better than the control group.

The Condition Medium used in this research contained 10% platelet-rich plasma (PRP) components, where PRP is also known to have a regenerative effect; however, many studies have shown that the content of growth factors in EVs is much more and varied compared to PRP, which only has cytokines, growth factors, and miRNA derived from platelets. Cytokines, growth factors, and miRNAs derived from PRP are typically associated with faster wound healing and angiogenesis, while EVs from Ad-MSCs often exhibit stronger immunomodulatory properties with a wider range of tissue regeneration capabilities. Thus, cellular communication pathway roles are played more by EVs derived from Ad-MSCs, so that the regeneration effect in this research was more due to the EVs than the PRP [51].

The healing of ligament/tendon tissue requires mechanical force. The application of tension to injured tendons can help the healing process by increasing fibroblast proliferation, migration, and collagen synthesis. Repairing tendons/ligaments in a stretched position makes fibroblasts and collagen fibers line up parallel to the direction of the tension force faster than when they are not stretched [52]. This basis is the reason that the application of EVs alone without repair or reconstruction cannot produce sufficient ligament tension to withstand the load.

EVs derived from Ad-MSCs have demonstrated great potential in therapeutic applications, especially in improving bone–ligament integration; thus, EVs seem to be able to be used as an adjuvant in ACL repair surgery for a better outcome. The isolation of EVs based on their density can be accomplished by ultracentrifugation with and without a density gradient. In these methods, the density differences between the medium and bioparticles, as well as among the bioparticles, provide opportunities for exosome separation. Ultracentrifugation (UC) is currently the gold standard in EVs isolation, and is widely used in laboratories, where roughly 56% of all EVs isolation is performed using this technique. However, in order that the use of EVs can be applied more widely, several obstacles need to be overcome immediately. One of the obstacles is to develop reliable and efficient protocols for the isolation and characterization of EVs. Developing standardized protocols for the isolation, purification, and characterization of exosomes is crucial for ensuring reproducibility and quality control across different production batches [53,54]. A comprehensive proteomic analysis of EVs did not detect the presence of major histocompatibility complex (MHC) I or MHC II. So far, there have been no reports of immunological reactions in experimental animals that have received EVs from humans. However, further research is still needed to validate the hypoimmunogenicity of these EVs for use in different diseases and at different doses [54]. The other potential limitations of EVs applications are because of its nanoparticle size and the electronegative charge, so it can be distributed widely and can cross the blood–epithelial barrier in vital organs (the blood–brain barrier and blood–gas barrier). This can be an advantage but also a disadvantage. The wide distribution means that, when given systemically, the EVs will be immediately absorbed into cells and organ parenchyma, so that they will be cleared in a short time from the blood vessels [55]. In this study, the EVs were given directly into the intratunnel, so that the local distribution increased, and the effects that occurred were expected to last longer than with systemic administration.

There are several limitations in this research. Human physiological conditions cannot be fully reproduced by animal models. The evaluations were carried out only in the first and sixth weeks, which might not reflect the actual conditions during the transition from the inflammatory phase to the proliferative phase, and the transition from the proliferative phase to the remodeling phase. Ideally, observations of the remodeling phase are carried out over a longer period. Another limitation is that we do not know the metabolism of the EVs after the intratunnel application related to the optimal concentration of active EVs molecules in the intratunnel, because no analysis was carried out on the inflammatory markers, anti-inflammatory markers in synovial fluid, and ACL histology.

## 5. Conclusions

In conclusion, EVs from adipose-derived MSCs could improve ligament–bone integration after ACL primary repair in rabbits by polarizing the M1 macrophages into M2 macrophages at the beginning of the inflammatory process, suppressing the MMP-3 levels and increasing the TIMP-1 levels, and increasing the number of type I collagen fibers at the bone–ligament interface, thereby having an impact on increasing the failure load after ACL primary repair.

## Figures and Tables

**Table 1:** Comparison of the iNOS, CD206, MMP-3, and TIMP-1 levels in the control and treated groups.

Marker	Group	Analysis			*p*-Value
		Mean ± SD	Median	Range (min–max)	
CD206 (pg/mL)	IA		210.74	144.13–264.65	0.126
	IIA		231.69	173.92–252.09	
	IIIA		196.45	107.05–208.53	
	IVA		228.53	214.92–295.89	
iNOS (ng/mL)	IA		0.31	0.20–0.56	0.068
	IIA		0.14	0.07–0.24	
	IIIA		0.15	0.01–0.20	
	IVA		0.13	0.08–0.15	
MMP-3 (ng/mL)	IA		2.19	1.49–2.52	0.012 *
	IIA		1.38	1.35–1.97	
	IIIA		1.65	1.27–1.76	
	IVA		0.81	0.71–0.93	
TIMP-1 (ng/mL)	IA	20.29 ± 10.528			0.029 *
	IIA	27.68 ± 11.657			
	IIIA	29.32 ± 14.604			
	IVA	54.63 ± 19.627			

Data are expressed as the mean ± standard deviation (SD) for the normal distribution data and expressed as the median with the interval range for the non-normal distribution data. Notation A indicates the group tested at the end of week 1. The one-way ANOVA test was used to determine the significant differences between the groups in the normal distribution data, while the Kruskal–Wallis test was used for the groups with the non-normal distribution data. * indicates a statistically significant difference.

**Table 2:** Comparison of type I collagen fibers in the control and treated groups.

Type I Collagen Fiber	Group	Analysis		*p* Value
		Median	Range (min–max)	
Longitudinal length in micrometer (μm)	IB	2.89	2.87–2.93	0.016 *
	IIB	3.11	3.05–3.15	
	IIIB	2.96	2.93–3.01	
	IVB	3.19	2.99–3.30	
Transversal diameter in micrometer (μm)	IB	1.81	1.89–2.04	0.016 *
	IIB	1.89	1.89–2.04	
	IIIB	1.79	1.77–1.87	
	IVB	1.99	1.90–2.07	

Data are expressed as the median with the interval range because the distribution was not normal. Notation B indicates the group tested at the end of week 6. The Kruskal–Wallis test was used to determine the significant differences between groups in the non-normal distribution data. * indicates a statistically significant difference.

**Table 3:** Comparison of type III collagen fibers in the control and treated groups.

Type III Collagen Fiber	Group	Analysis		*p* Value
		Mean	Standard Deviation	
Longitudinal length in micrometer (μm)	IB	3.20	0.20	0.367
	IIB	3.29	0.18	
	IIIB	3.41	0.06	
	IVB	3.31	0.07	
Transversal diameter in micrometer (μm)	IB	1.96	0.17	0.243
	IIB	2.02	0.13	
	IIIB	2.14	0.07	
	IVB	2.09	0.05	

Data are expressed as the mean ± standard deviation (SD) because of the normal distribution. Notation B indicates the group tested at the end of week 6. The one-way ANOVA test was used to determine the significant differences between the groups in the normal distribution data.

## Data Availability

The research data from this study can be requested from the author via email: andreyanuar@yahoo.com.

## Abbreviations

The following abbreviations are used in this manuscript:

ACL	Anterior Cruciate Ligament
ELISA	Enzyme-Linked Immunosorbent Assay
iNOS	Inducible Nitric Oxide Synthase
CD206	Cluster of Differentiation 206
MMP-3	Matrix Metalloproteinase 3
DNA	Deoxyribonucleic Acid
mRNA	Messenger RNA
lncRNA	Long Noncoding RNA
miRNA	MicroRNA
